# Authors- the most dangerous pressure group

**DOI:** 10.12669/pjms.306.6465

**Published:** 2014

**Authors:** Shaukat Ali Jawaid

Research scientists, academicians including faculty members in medical institutions under great pressure to publish papers to get promotions and higher grade indulge in all sort of scientific misconduct all over the world. Some of these include plagiarism, salami slicing, ghost authorship, falsification of data, fabrication of data and at times duplicate submissions etc. The revolution in information technology and tremendous developments which has taken place in the scientific publishing has though eased the job of editors of biomedical journals but it has not reduced their problems which in fact have further multiplied. ^1^^-^^2^

The authors are so impatient that some of them want their manuscripts to be processed and published in the shortest possible time. Given below are some of the e mails which were received during the last few months from the authors which show their urgency:

1. I have been offered Residency in United States. I have got the Visa and also booked my seat but I urgently need one published paper in an Impact Factor journal. If you help me, I will be prepared to pay the Fee, as money is not a problem.

2. I have to defend my Ph.D Thesis and before the exam, I need one published paper in Impact Factor Journal. Willing to pay the desired fee if the paper is published in the next issue.

3. When is your next issue of the journal due as I wish to submit a paper for publication?

4. I have many research papers. I can submit five to six papers every month and if you enter into an agreement to publish two of my papers in every issue, we can negotiate the publication charges but would expect some discount keeping in view the large number of papers.

5. I forgot to get Ethics Committee approval but I need this paper to be published quickly to be considered for promotion by the university. Need your help and a sympathetic consideration.

6. I am submitting a research paper and it does not need any peer review as Prof…….. has already seen it and approved its publication. Please include it in the issue under printing and let me know the publication charges so that I can arrange it immediately.

7. My university accepts only those papers for academic credit which are published in journals covered by ISC, Web of Sciences with Impact Factor. I have three papers. If you can publish them in the previous issues and include them in the online edition, I can claim the credit. Publication charges no problem. Need your help.

8. We have some problems with the institutional ethics committee because of professional jealousy and ------- politics. Can you help us accept our papers without Ethics Committee approval? We assure you there won’t be any scientific misconduct in our studies. 

9. In the paper published in your current issue, the research was done by me but my supervisor sent you the manuscript putting his name at No. 1 in the authors list. Kindly do justice to me and put my name as No. 1 and if possible remove the name of my supervisor, as I am no more working there.

10. I made a mistake of submitting my research study to your very low quality journal. By rejecting my paper, you have proved that authors must know and have friendship with the editors if they wish to get their manuscripts published.

This is not all. If you look at the other e mails with uncharitable remarks and comments from those authors whose manuscripts are rejected, their comments for the Reviewers, one feels very sad. Instead of learning and improving their manuscripts benefitting from the reviewers comments and suggestions, they indulge in un-necessary correspondence and uncalled for allegaitons. In fact editing a quality peer reviewed medical journal with financial and human resource constraints is a very frustrating and stressful job and the editors have to face numerous problems and different pressure groups. 3^-^^5^ Talking about the problems faced with the authors, Dr. Ahmad Baddar a distinguished medical editor who edited Journal of Ayub Medical College for many years, once informed me that he does not talk to the authors. He just communicates with them through e mail. At first I thought it was a bit rude attitude but over the years I have realized that it is the more sensible approach because it is not easy to make the authors understand the problems the editors have to face. This also reminds me of the keynote speech delivered by Mr. Tim Albert an eminent medical journalist from UK who while making a presentation as guest speaker at the EMMJ4 Medical Journals Conference organized by Eastern Mediterranean Associaton of Medical Editors (EMAME) at Bahrain in 2008 had remarked that “authors are the most dangerous pressure groups the editors have to face”^.^^6^

However, there is another category of authors who are keen learners. They utilize the opportunity provided by the editorial team and reviewers comments to improve the quality of their manuscripts. These are the one who become much wiser and later also serve as Reviewers on being picked up by the Editors. Some of them are even honoured by inviting them to join the Editorial Board of various journals.

Gift authorship is one of the scientific misconduct which is highly prevalent every where and Pakistan is no exception. Gift authorship is mostly offered to the seniors, head of the department or institutions, to friends on reciprocal basis. At times this is not a gift but it is “authorship for sale”. Once the manuscript has been accepted for publication, the authors take money from others who wish to have some published papers and then ask the editors to make changes in the authors, add some authors and delete some. ^7^ Though gift authorship is quite common but keeping in view the sensitive nature, it is difficult to find out its true prevalence.

Over the years misconduct has crept into research in different forms. A recent study from India reports that out of 192 filled questionnaires from nine centers, 27 were excluded from final analysis. Hence out of the total remaining 155 Reponses in 86, (85.1%) instances the person offered gift authorship was a senior. Fifty one (32.9%) knew of their colleagues who had sliced up their studies to created multiple papers from the single study. 8

These are some of unethical practices which the editors have to check with courage and determination. The Editors are supposed not to betray their readers and tell the truth, promote scientific, evidence based medicine and check publication of un-authenticated, wrong or doctored information. Even the recently revised guidelines on authorship by International Committee of Medical Journal Editors (ICMJE) has proposed the four criteria to which all authors are now accountable. ^9^

Based on the personal experience one can offer some suggestions to the editor colleagues to follow and protect their as well their journal’s integrity and credibility:

- Be careful of too many submissions from the same e mail. Some agency can be at work.
- Do not entertain submissions from professional business, commercial groups; instead always communicate directly with the authors.
- Do not process any manuscript unless it is accompanied by Ethics Committee/Institutional Review Board (EC/IRB) approval. Even if ethics committee approval is not needed, let the authors provide an exemption letter issued by the EC/IRB.
- Check the menace of Gift authorship as far as possible. Refuse to change, add or delete authors from the list of original submission or those which are mentioned on the letter of undertaking at the time of submission. However, if the authors insist ask them to get it approved first from their institution and then from the Ethics Committee/Institutional Review Board which had approved the study protocol.
- Have a good Peer Review system in place. Even if the manuscript has been reviewed by the reviewers suggested by the authors, there is no harm in it but carefully Review their Reviews and if need be seek help and assistance of other reviewers.
- In case you suspect gift authorship and the authors insist on retaining all the listed authors, you can always politely refuse to process such manuscripts and ask the authors to submit it to some other journal.
- Maintain complete record of correspondence with authors, peer review for at least two years.
- Always screen all the manuscripts for plagiarism before processing them further.
- Local reviewers are best at detecting salami publications.

Finally the editors should not expect to find a solution to all these problems in the very near future. However, the solution lies in improving the knowledge of medical professionals regarding publication ethics besides strengthening the mechanism to identify and weed out scientific misconduct. We have to cover a lot more distance in this academic journey in the field of scientific publishing.
